# Predictors of Successful Medical Management With Methotrexate in Unruptured Tubal Ectopic Pregnancy

**DOI:** 10.7759/cureus.31923

**Published:** 2022-11-26

**Authors:** Alokananda Ray, Ankita Gaur, Sarita Kumari

**Affiliations:** 1 Obstetrics and Gynaecology, Tata Main Hospital, Jamshedpur, IND

**Keywords:** receiver operating characteristic (roc) analysis, successful treatment outcome, serum beta hcg, unruptured tubal ectopic pregnancy, methotrexate

## Abstract

Introduction

Medical treatment with methotrexate (MTX) is a safe and effective alternative to surgery in carefully selected cases of ectopic pregnancies diagnosed early prior to rupture.

Aim

To determine the optimal pre-treatment levels of beta human chorionic gonadotropin (𝛽-hCG) and its changing trends most likely to have a successful outcome with medical management.

Material and methods

A prospective observational study was conducted in a tertiary teaching hospital from December 2018 to May 2021. “Single-dose” MTX regime was used for medical management of ectopic pregnancy in patients fulfilling the selection criteria. The 𝛽-hCG levels were recorded at baseline and on day 4 and day 7 of MTX injection. Thereafter, at weekly intervals till complete resolution or surgical intervention due to failure of medical management. In addition, receiver operating characteristic (ROC) curve analysis for a pre-treatment 𝛽-hCG cut-off value and changing trends in post-treatment 𝛽- hCG levels most likely to have a successful outcome with MTX treatment were determined.

Results

Fifty patients fulfilling the inclusion criteria were included in the study, with successful medical management in 33 (66%). The mean pre-treatment 𝛽-hCG* *levels in women with successful medical management were 3270.97 (+/- 901) compared to 5249.17 (+/-808.02) for those with treatment failure (p=0.00001). The mean 𝛽-hCG level in the failed treatment group was significantly higher on day 4 than the pre-treatment levels (6742.56 +/- 572 vs. 5249.17+/- 808.02; p<0.05). Inadequate reduction of 𝛽-hCG level on day 7 (<15% of day 4 levels) requiring repeat dosage of MTX was more likely to have an unsuccessful outcome (p=0.00001). The area under curve (AUC) value of 0.905 (95% CI: 0.814-0.996) depicted that pre-treatment 𝛽-hCG level of 4000 mIU/ml taken as the cut-off value was able to predict levels ≤4000 mIU/ml had a greater likelihood of successful outcome with MTX, having a sensitivity of 84.5%, specificity of 83.3%, positive predictive value (PPV) of 90.3%, and negative predictive value (NPV) of 75% (p< 0.05). Demographic variables or previous clinical history, considered risk factors for ectopic pregnancy, did not affect the outcome of medical management in this study.

Conclusion

Medical management of ectopic pregnancy is a viable first-line treatment option in carefully selected patients. In this study, the most important predictors for the successful outcome of medical management were the pre-treatment β-hCG levels and their fall on day 4 and day 7 after MTX therapy.

## Introduction

Ectopic pregnancy, a significant cause of morbidity and mortality in the first trimester, is a clinical condition when the developing embryo implants at a site other than the endometrium of the uterine cavity. The most common extra-uterine location is the fallopian tube, which accounts for nearly 95-98% of all ectopic gestations. [1] The incidence of ectopic pregnancy has been reported as 0.25%-2%. [2,3] The increase in the incidence in recent years is attributed to better awareness and advanced techniques for diagnosing ectopic pregnancy coupled with an increasing prevalence of risk factors for ectopic pregnancy, such as pelvic inflammatory disease (PID) and assisted reproductive technology (ART) [3].

It is one of the few clinical conditions that can be managed either by medical treatment or by surgery. Medical treatment with methotrexate (MTX) is a safe and effective alternative to surgical management in properly selected cases diagnosed early prior to rupture [4]. However, the predictors for the outcome of MTX therapy in ectopic pregnancy lack well-defined boundaries with regard to its success or failure.

Therefore the study was undertaken with the aim to evaluate the outcome in patients undergoing medical management with methotrexate in un-ruptured tubal ectopic pregnancy and to determine the optimal pre-treatment and post-treatment trends in beta-human chorionic gonadotropin (𝛽-hCG) levels most likely to have a successful outcome with medical management.

## Materials and methods

This prospective observational study was conducted from December 2018 to May 2021 in the department of Obstetrics and Gynaecology of a tertiary teaching hospital in Jharkhand, India. The study population included patients who presented to the hospital with clinical, biochemical, and ultrasound evidence of un-ruptured tubal ectopic pregnancy. It fulfilled the following inclusion criteria: 1) Serum β-hCG (<7000 mIU/mL), 2) trans-vaginal ultrasound (TVS) showing an extra-uterine tubal gestational sac of size <3.5cm, 3) hemodynamically stable patient, 4) in the presence of a fetal pole, absence of fetal cardiac activity, 5) no contraindication to MTX therapy, and 6) patient willing for compliance to follow-up. Patients having one or more of the following features were excluded from the study: 1) severe abdominal pain, 2) hemodynamically not stable, 3) serum β-hCG (>7000 mIU/mL), 4) gestational sac size >3.5 cm/presence of fetal cardiac activity, and or 5) ruptured tubal ectopic pregnancy.

The sample size (n) was calculated using the formula: n = Z 2 x (p) x (1-p) / L 2. Taking a 95% CI, relative error (L) as 5%, the proportion of the population with the disease (p), and prevalence of ectopic pregnancy as 3%, and the calculated sample size for obtaining results of statistical significance was 44.7. Considering a drop-out rate of 10%, 50 patients were included in the study group.

The diagnosis of tubal ectopic pregnancy was made by using TVS with the detection of an extra-uterine gestational sac in the fallopian tube and by measurement of abnormally low doubling time of serial 𝛽-hCG at 48 hours intervals. All women selected for medical management as per the defined inclusion criteria gave their informed written consent before initiation of the treatment.

The demographic data, history of previous ectopic pregnancy, PID, ART procedures, gestational age at diagnosis, presenting signs and symptoms, ultrasound findings, general, physical, systemic, and gynecological examination findings, and the need for hospital admission were documented. All the patients in the study group received injection MTX as a single 50 mg/m2 dose by intramuscular route. Before the first dose of MTX, women were screened with a complete blood count (CBC), liver function tests, serum creatinine, and blood group Rh typing. In addition, women were advised to stop supplemental folic acid therapy.

The 𝛽-hCG levels were measured and recorded at baseline and on day 4 and day 7 of MTX injection. Thereafter, serial serum 𝛽-hCG was obtained at weekly intervals. When 𝛽-hCG levels failed to decrease by at least 15% from the day 4 level, the second dose of MTX injection (50 mg/m2) was given on day 7. A maximum of two doses were considered before declaring it a failed medical management. In all cases receiving MTX, the serum 𝛽-hCG levels were monitored until they fell below the cut-off value of <5 mIU/ml. The time of resolution of ectopic pregnancy was defined as the total number of days from the beginning of treatment until 𝛽-hCG levels tested negative (<5mIU/ml). Failure of medical management was defined as the need for surgical intervention. Indications of surgery were classified as 1) insufficient fall or rise of serum 𝛽-hCG level, 2) rupture of tubal pregnancy, and 3) persistent or worsening clinical symptoms related to tubal pregnancy.

Data analysis was done using SPSS IBM version 21.0. The qualitative variables were described in the form of proportions, and quantitative variables were described in terms of mean, median, range, and SD. The significance of the difference in means was calculated using the independent t-test, and the difference in proportions was calculated with the Chi-square test. Receiver operating characteristic (ROC) curve analysis was performed to derive a cut-off pre-treatment level of β-hCG likely to predict the successful outcome with MTX treatment. The significance of the p-value was taken as p< 0.05.

The study was approved by the Research Ethical Committee of the hospital.

## Results

Fifty patients fulfilling the inclusion criteria were included in the study group. The mean (SD) age of the study population was 29 (±2.8) years, ranging from 21 to 35 years. Thirty (60%) patients were primigravidas, and 28 (56%) were from rural areas. The last childbirth prior to the index ectopic pregnancy was >5 years in 18/20 (90%) multiparous women in the study group.

Twenty-two participants (44%) had been treated for infertility at some point during their obstetric carrier, and 5 (10%) had undergone ovulation induction or ART procedure in the index ectopic pregnancy. Thirteen patients (26%) had a previous history of PID, seven (14%) had a prior history of ectopic pregnancy, and two (4%) had undergone tubal ligation for fertility control in the past.

The presenting chief complaint was amenorrhea in 25 (50%), vaginal bleeding in 11 (22%), abdominal pain in five (10%), and amenorrhea pain with bleeding in nine (18%) women. The mean gestational age at detection of ectopic pregnancy was six weeks two days (6W2D), (range 5W2D-8W3D). The baseline 𝛽-hCG level was between 1000 and 4000 mIU/ml in 31 (62%) and 4001-7000 mIU/ml in 19 (38%) women. In transvaginal sonography, the gestational sac was <3 cm in 45 (90%) and 3-3.5 cm in five (10%) study population patients.

All the patients in the study group received an MTX injection of 50 mg/m2 as a single intra-muscular dose. The 𝛽-hCG levels were measured and recorded at baseline and on day 4 and day 7 of MTX injection. Thereafter, serial serum 𝛽-hCG was obtained at weekly intervals. When 𝛽-hCG levels failed to decrease by at least 15% from the day 4 level through day 7, the second dose of MTX injection (50 mg/m2) was given with weekly 𝛽-hCG estimation. A maximum of two doses were considered before declaring it as a failed medical management unless the patient required a surgical intervention prior to that as per defined indications. In all patients, the serum 𝛽-hCG levels were monitored until they fell below the cut-off value of <5 mIU/ml. This study documented successful medical management in 33 patients (66%). The average time of resolution for ectopic pregnancy (defined as the number of days from the first dose of MTX to 𝛽-hCG <5 mIU/ml) was 30.6 days (range 14-42 days) for a single dose of MTX and 47.2 days (range 21-63 days) for two doses of MTX. 

Analysis of outcome in patients with respect to the pre-treatment 𝛽-hCG levels showed that successful medical management was noted in 5/5 (100%) for 𝛽-hCG levels ≤3000 mIU/ml, 27/28 (96%) for 𝛽-hCG levels ≤3500 mIU/ml, and 28/31 (90%) for 𝛽-hCG levels ≤4000 mIU/ml. For women with 𝛽-hCG levels between 4001 and 5000, medical management was still successful in 4/7 (57%) women. However, above 5000 mIU/ml, the success of medical management sharply declined to 1/12 (8.3%) (Table 1).

**Table 1:** Pre-treatment 𝛽-hCG levels and outcome to medical management. MTX: Methotrexate; 𝛽-hCG: Beta human chorionic gonadotropin.

Range of 𝛽-hCG	Total number n (50)	Successful with MTX n (33)	Required surgery n (17)
1000-2000	3	3	0
2001-3000	2	2	0
3001-4000	26	23	3
4001-5000	7	4	3
5001-6000	11	1	10
6001-7000	1	0	1

Women with lower levels of pre-treatment 𝛽-hCG had a greater likelihood of successful outcomes with medical management. The mean pre-treatment 𝛽-hCG levels in women with successful medical management were 3270.97 (+/- 901) compared to 5249.17 (+/-808.02) for those with treatment failure, this was statistically significant (p<0.00001). 𝛽-hCG levels in women with unsuccessful outcomes were 1.6 times higher than those with successful outcomes, and the difference in the mean was >2SD.

Five of 31 women with 𝛽-hCG ≤4000 mIU/ml required repeat injections of MTX due to inadequate fall of 𝛽-hCG level on day 7 (medical management failed in two of them). Fifteen of 19 patients with 𝛽-hCG >4000 mIU/ml required repeat injections of MTX, and medical management failed in 14 of them. The rest of the 30 patients (26 with 𝛽-hCG ≤4000 mIU/ml and four with 𝛽-hCG >4000 mIU/ml) received a single dose of MTX, with treatment failure in one patient. In this patient, 𝛽-hCG was <4000 mIU/ml, and she underwent salpingectomy after a single dose of MTX due to worsening symptoms. Patients with inadequate reduction of 𝛽-hCG level on day 7 (<15% of day 4 levels) requiring repeat dosage of MTX were more likely to have an unsuccessful outcome (p=0.00001) (Table 2).

**Table 2:** Lower pre-treatment 𝛽-hCG level, with effective fall on day 7 (≥15% of day 4 levels) after the primary injection of MTX, had a significantly higher likelihood of successful outcome with medical management. MTX: Methotrexate; 𝛽-hCG: Beta human chorionic gonadotropin.

Variable		Outcome		Chi-square test (P-value)
		Successful	Unsuccessful	
Pre-treatment 𝛽-hCG	Total numbers			
1000-4000 mIU/ml	31	28	3	21.5067 (P<0.00001)
4001-7000 mIU/ml	19	5	14	
Doses of MTX injections				
One	30	29	1	31.4 (P=0.00001)
More than one	20	4	16	

Twenty (40%) patients had higher 𝛽-hCG levels than the baseline pre-treatment level on day 4 of MTX treatment. The mean 𝛽-hCG level in the failed treatment group (n=17) was significantly higher on day 4 than on the initial day of MTX treatment (6742.56 +/- 572 vs. 5249.17+/- 808.02; p <0.05).

In 17 women (34%), medical management failed, and surgical intervention was required. Indications for surgery included plateauing or rising 𝛽-hCG levels despite two doses of MTX in seven patients, worsening symptoms of abdominal pain in five, and increased adnexal mass size with pain in three and tubal rupture with haemo-peritoneum in two. Of the 17 patients undergoing surgery, nine had laparoscopic salpingectomy, three had laparotomy with salpingectomy, and five underwent laparoscopic salpingotomy or tubal milking procedure with tubal preservation. Tubal preservation was possible for all 33 patients with a successful medical management outcome.

The test of significance for the various demographic or clinical variables (age, parity, history of PID, previous history of ectopic pregnancy, and history of infertility) and their association with the outcome of medical management was found to be statistically not significant (age [p=0.827], parity [p=0.180], history of PID [p=0.775], history of infertility [p=0.146], history of previous ectopic pregnancy [p=0.713]). Demographic variables and previous clinical history, considered risk factors for ectopic pregnancy, did not affect the outcome of medical management in this study.

**Figure 1:**
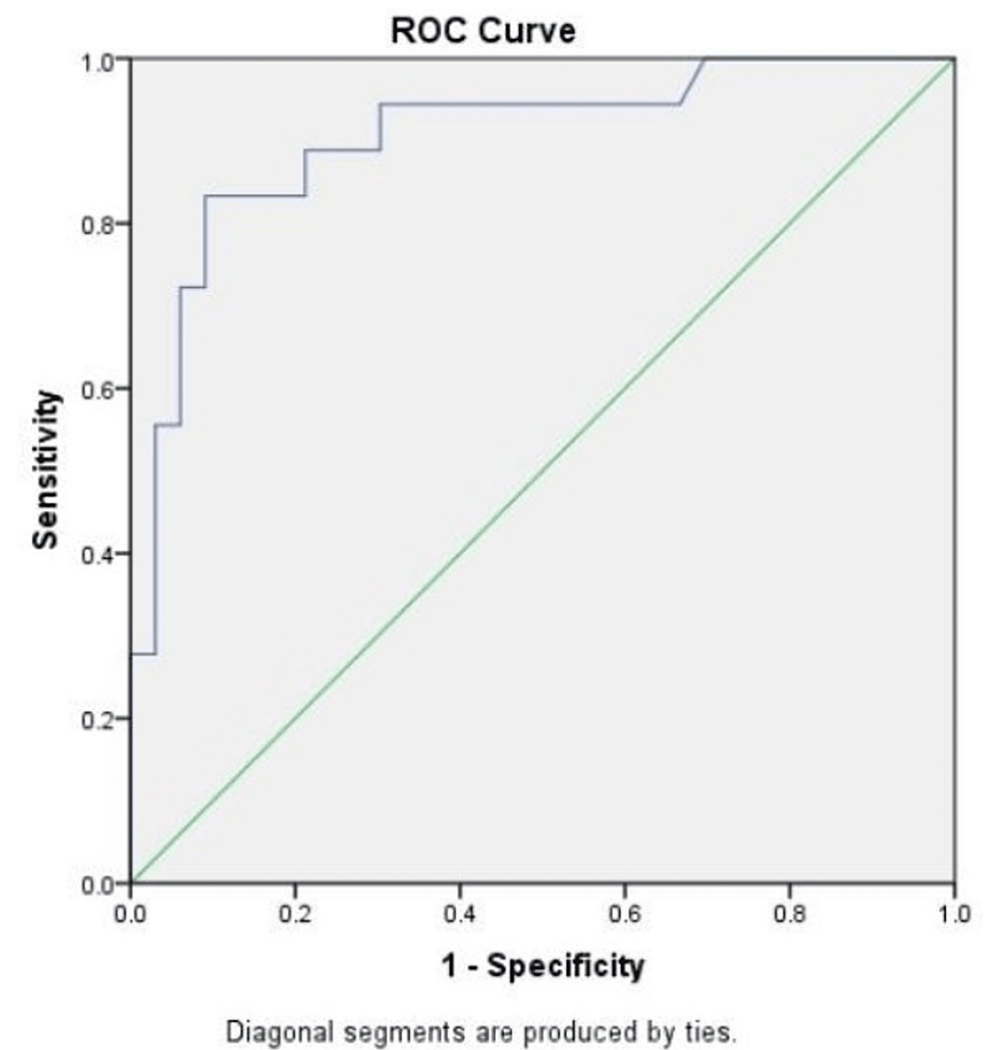
ROC curve analysis for the prediction of outcome by pre-treatment 𝛽-hCG levels. A total of 4000 mIU/ml was taken as the cut-off for the outcome prediction, and the ROC curve was plotted (n=50). The AUC, with 95% CI, was calculated as 0.905 (95% CI: 0.814-0.996). ROC curve: Receiver operating characteristics curve; AUC: Area under curve; 𝛽-hCG: Beta human chorionic gonadotropin.

ROC curve analysis was done to evaluate the performance of pre-treatment 𝛽-hCG levels as a diagnostic tool to predict the successful outcome of medical management for ectopic pregnancy (Figure 1). The area under the curve (AUC), with 95% CI, was calculated as 0.905 (95% CI: 0.814-0.996). The AUC value of 0.905 depicted that pre-treatment 𝛽-hCG level of 4000 mIU/ml taken as the cut-off value was able to predict that 𝛽-hCG levels of ≤4000 mIU/ml had a greater likelihood of successful outcome after medical management, with a sensitivity of 84.9%, specificity of 83.3%, positive predictive value (PPV) of 90.3%, and negative predictive value (NPV) of 75%. This was found to be statistically significant (p<0.05).

## Discussion

Ectopic pregnancy occurs in around 1-2% of pregnant women and is an important cause of morbidity and mortality in the first trimester of pregnancy. It can also seriously compromise future fertility in the woman and hence is an important clinical entity to diagnose and manage [2-3]. The cornerstone for early detection of ectopic pregnancy prior to rupture and worsening signs and symptoms are a high index of suspicion, trans-vaginal ultrasonography with detection of the ectopic gestation, and serial 𝛽-hCG assays showing abnormal doubling time or plateauing levels [5]. Any pregnant woman can suffer an ectopic pregnancy; however, some risk factors, especially those causing damage to the fallopian tubes, can predispose her to ectopic pregnancy [6-8]. Hence, knowledge of these risk factors (such as the previous history of ectopic pregnancy, tubal surgery including sterilization, PID, sexually transmitted diseases (STD), treatment for infertility by ART, or cigarette smoking) can help identify women who might benefit from close monitoring, with early detection and management of ectopic pregnancy. In this study, 5 (10%) had undergone ovulation induction or ART procedure in the index ectopic pregnancy, 13 (26%) had a previous history of PID, seven (14%) had a prior history of ectopic pregnancy, and two (4%) had undergone tubal ligation in the past. A high degree of suspicion and close monitoring helped diagnose these cases early before rupture, enabling medical management.

Medical management with MTX, a folic acid antagonist, has been shown to achieve outcomes comparable to surgery in carefully selected cases of ectopic pregnancies. Literature suggests that medical treatment protocols with MTX are widely accepted as the primary treatment for ectopic pregnancy, and surgery is indicated only in cases of suspected tubal rupture or when MTX is contraindicated [9,10]. Ideally, a candidate for medical management with MTX should be hemodynamically stable, have no severe or worsening symptoms of abdominal pain, have no contraindications to MTX therapy, and have good access to follow-up until the ectopic pregnancy has resolved [4]. All 50 cases included in our study met these selection criteria.

The two commonly used MTX treatment regimens are multiple-dose and single-dose therapy. The multiple-dose protocol alternates MTX (1 mg/kg) with leucovorin rescue (0.1 mg/kg) therapy and 𝛽-hCG assay on the days scheduled for MTX injection prior to administering it. MTX is continued until 𝛽-hCG falls by 15% from its peak concentration [9,11]. With the single-dose regimen, MTX is administered at a dose of 50 mg/m2 with 𝛽-hCG assay on day 4 and day 7. The term "single dose" is a misnomer because additional doses of MTX can be administered when the response is inadequate [12]. Single-dose MTX appears to be effective, does not require leucovorin rescue, and has better patient tolerability and compliance [13].

In both single- and multiple-dose MTX treatment regimens, once 𝛽-hCG levels meet the criteria of a 15% fall from peak levels, MTX is withheld, and 𝛽-hCG levels are monitored at weekly intervals till it becomes undetectable. Complete resolution of an ectopic pregnancy usually takes between 2 and 8 weeks, depending on pre-treatment 𝛽-hCG levels [11-12]. If declining levels of 𝛽-hCG rise again or plateau, the diagnosis of failed medical therapy and a persistent ectopic pregnancy is made. In our study, all patients received the "single dose" regimen of MTX (50 mg/m2) as per protocol, with no documented side effects to the drug in any patient.

Our study's overall success rate for medical management enabling tubal preservation was 33/50 (66%). Several studies have reported a higher success rate of 75%-95% [14,15]. We attribute the lower success rate in our study to the inclusion of women with higher pre-treatment 𝛽-hCG levels. Studies have reported two to three times higher pre-treatment 𝛽-hCG levels in the group with the unsuccessful outcome with MTX [13,16]. In our study, the mean pre-treatment 𝛽-hCG levels in women with failed medical management were 5249.17 (+/-808.02) compared to 3270.97 (+/- 901) in those who successfully responded to the MTX therapy. This was statistically significant (p=0.00001). Beta-hCG levels in women with unsuccessful outcomes were 1.6 times higher, and the difference in the mean was >2SD. Successful medical management was noted in 5/5 (100%) women with 𝛽-hCG levels ≤ 3000mIU/ml and 28/31 (90.3%) with ≤4000 mIU/ml. However, above 5000 mIU/ml, the success of medical management sharply declined to 1/12 (8.3%). For women with 𝛽-hCG levels between 4001 and 5000, the medical management was successful in 4/7 (57%) women, which is comparable to a previous study, stating a 50% success rate in women with hCG levels >4000 mIU/ml [17]. The same study also concluded that MTX might be considered a viable treatment option for asymptomatic, hemodynamically stable women with ectopic pregnancies regardless of their initial serum β-hCG levels or adnexal mass sizes. However, they may require a longer duration of follow-up, repeated doses of MTX, and an increased likelihood of the unsuccessful outcome of medical management requiring emergency surgical intervention [17].

The average time of resolution for ectopic pregnancy was 30.6 days (range 14-42 days) for a single dose of MTX and 47.2 days (range 21-63 days) for two doses of MTX in our study. This is comparable to other studies reporting complete resolution after MTX therapy, usually between 2 and 3 weeks, which can take as long as 6-8 weeks when pre-treatment 𝛽-hCG levels are in higher ranges [11-12].

In our study, ROC analyses for an AUC value of 0.905 (95% CI: 0.814-0.996) depicted that pre-treatment 𝛽-hCG level of 4000 mIU/ml taken as the cut-off value was able to predict that pre-treatment levels of ≤4000 mIU/ml had a greater likelihood of successful outcome after medical management, with a sensitivity of 84.9%, specificity of 83.3%, positive predictive value (PPV) of 90.3% and negative predictive value (NPV) of 75%. This was found to be statistically significant (p<0.05). In another recent study, ROC analysis with a pre-treatment 𝛽-hCG cut-off value of 3924 IU/L deduced a 76.19% sensitivity and 62.5% specificity with a treatment success rate of 75% [13].

Several studies have reported that in 26-60% of women who receive MTX, there is a transient increase in the 𝛽-hCG concentration on day 4 compared to pre-treatment levels. This is because of the effect of MTX on trophoblast cells [17,18]. In our study, 20 (40%) patients had higher 𝛽-hCG levels on day 4 of MTX treatment. The mean 𝛽-hCG level in the failed treatment group (n=17) was significantly higher on day 4 than on the day of MTX injection (6742.56 +/- 572 vs. 5249.17 +/- 808.02; p<0.05). A recent study based on the ROC analysis has reported that a decrease in the 𝛽-hCG level of at least 8.2% between day 1 and day 4 was associated with an 88.6% probability of successful outcomes without any further intervention [17].

Demographic variables (age, parity) or known risk factors for ectopic pregnancy (history of ectopic pregnancy, PID, infertility) did not affect the outcome of medical management in our study. Lower pre-treatment 𝛽-hCG levels, further falling on the fourth and seventh day of the primary injection of MTX therapy, were more likely to have a successful outcome. This observation is comparable to other studies [17-19].

## Conclusions

In this study, the most important predictors for the successful outcome of medical management were lower pre-treatment 𝛽-hCG levels (≤4000 mIU/ml), the absence of its rising trend on post-treatment day 4, and a further decrease by at least 15% (of day 4 levels) on post-treatment day 7, in response to a single dose of MTX.
